# Correction: *Randomised controlled trial to determine the efficacy and safety of prescribed water intake to prevent kidney failure due to autosomal dominant polycystic kidney disease (PREVENT-ADPKD)*

**DOI:** 10.1136/bmjopen-2017-018794corr1

**Published:** 2018-08-05

Wong ATY, Mannix C, Grantham JJ, *et al*. Randomised controlled trial to determine the efficacy and safety of prescribed water intake to prevent kidney failure due to autosomal dominant polycystic kidney disease (PREVENT-ADPKD). *BMJ Open* 2018;8:e018794. doi: 10.1136/bmjopen-2017-018794.

This article was previously published with an error in the author list.

Co-author Eswari Vilayur surname was incorrectly spelt as Vliayuri.

